# Progress in the Preparation and Characterization of Convex Blazed Gratings for Hyper-Spectral Imaging Spectrometer: A Review

**DOI:** 10.3390/mi13101689

**Published:** 2022-10-07

**Authors:** Huang Li, Xiaoqiang Peng, Chaoliang Guan, Hao Hu

**Affiliations:** 1 College of Intelligence Science, National University of Defense Technology, Changsha 410073, China; 2 Laboratory of Science and Technology on Integrated Logistics Support, National University of Defense Technology, Changsha 410073, China; 3 Hu’nan Key Laboratory of Ultra-Precision Machining Technology, Changsha 410073, China

**Keywords:** spectral imaging spectrometer, convex blazed grating, spectral range, spectral resolution, fabrication, characterization

## Abstract

Convex blazed gratings, which can effectively broaden the spectral range and improve spectral resolution, have gradually evolved into a crucial optical component for lightweight and compact imaging spectroscopy instruments. Their design, processing, and testing involve multidisciplinary interdisciplinary scientific issues, and they continue to be a major area of research in imaging optics applications. This paper summarizes the effects of various grating groove shapes and structural parameters on the spectral range and diffraction efficiency of convex blazed gratings, after providing a brief introduction to the typical functions and applications of convex blazed gratings. Firstly, the latest progress in typical processing methods for convex blazed gratings is reviewed. It focuses on the current fabrication processes and reviews their capabilities in creating convex blazed gratings from three main types of technologies, namely ultra-precision machining, high-energy density beam processing, and chemically assisted fabrication processes. Secondly, the adaptability of the manufacturing process for convex blazed gratings on different scales is summarized, analyzing the adaptation of current procedures to various grating fabrication scales and their bottlenecks. Finally, the characterization methods and future feasible characterization methods for convex blazed gratings are reviewed. The development trend of efficient and precise preparation of convex blazed gratings is pointed out.

## 1. Introduction

Due to their exceptional performance, microstructured functional surfaces are frequently used in advanced science and industry; yet, the surface quality of the production procedure limits their performance [1]. Fresnel lenses and diffractive optical elements (DOEs), which can be utilized to enhance optical performance, such as beam forming or aberration correction while reducing the weight of optical systems, are typical examples of microstructured optics applications. Diffraction gratings [2,3,4] are optical components that have a periodic, subwavelength-scale range of optical functional structures that can spatially modulate the amplitude or phase of the incident light. They are extensively used in a variety of industries, including spectral instruments, laser modulation, optical metrology, information processing, thin-film optics, and polarization optics.

**Figure 1:**
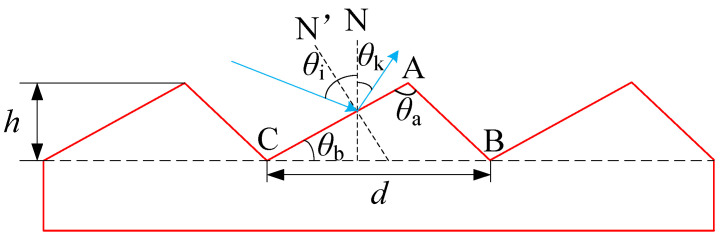
Working principle of reflective blazed grating.

Wood [5] invented the “blazed” grating technique in 1910 to increase the diffraction efficiency of the grating. This technique alters the energy distribution of light at each diffraction order by altering the form of the grating groove. The grating can transfer spectral energy from the zeroth order to the desired order of the spectrum to achieve the “blaze” of the order if the grooved surface of the grating is not parallel to the grating’s normal, i.e., if there is a small angle *θ*_b_ (blaze angle, as shown in Figure 1) between the two. The wavelength corresponding to the maximum light intensity is the blazed wavelength. The design of the blaze angle permits the application of the grating to a specific order of the spectrum within a specific wavelength range. In Figure 1, N represents the grating normal, N’ represents the normal of the grating groove (i.e., the normal of the blazed surface), *θ*_i_ represents the angle of incidence of the light wave, and *θ*_k_ represents the angle of diffraction.

Planar gratings are often used in monochromators, concave gratings are often used in portable spectrometers, and convex grating spectrometers are more often used in aerospace high-resolution hyper-spectral imaging systems because of their symmetrical structure, total reflection, and large image field. In 1973, Offner [6] came up with the idea of a concentric three-mirror imaging system that could make high-quality images in a circular area. Mertz [7] came up with the idea of the Offner spectrometer by putting a convex diffraction grating in place of the Offner structure’s convex reflector. In 1999, M. P. Chrisp et al. [8] used two small concave mirrors to replace a concave primary mirror that was centered on the convex grating. This made it possible to make an Offner spectrometer that was small and portable. Shen Y H et al. [9] optimized the structure of the Offner imaging spectrometer to improve the diffraction efficiency of the first order of the convex blazed grating by 27%. Xue Q et al. [10] made a convex grating imaging spectrometer with a spectral resolution of more than 0.5 nm. Hu Y et al. [11] introduced a construction method for designing a spectrometer with variable spectral resolution and multiple off-axis convex gratings. Zhu J. et al. [12,13] made a small VNIR Wynne–Offner spectrometer with a long slit. It has a smaller structure and better image quality than the classic Offner spectrometer. The hyper-spectral imager designed by Wang B et al. [14] adopts the Wynne–Offner structure to achieve high imaging quality, low smile, and keystone. The Offner imaging spectrometer is known for its large field of view, high image quality, small size, and small spectral curve, which is used a lot in geology, environmental monitoring, disaster warning, aerospace, and other fields [15,16,17].

In *Light: Science & Applications*, published in 2017, Reimers J et al. [18] showed that Offner–Chrisp freeform imaging spectrometers can correct both blur aberrations and distortions. This makes them work well in many applications. With freeform surfaces, it is easier to make compact and well-corrected Offner spectrometer structures, and De Clercq C et al. [19] made a new ELOIS (Enhanced Light Offner Imaging Spectrometer) spectrometer with freeform gratings based on Offner spectrometer convex gratings. Wei L et al. [20] optically simulated the Offner–Chrisp imaging spectrometer by choosing XY polynomial surfaces from three freeform surfaces as mirrors. This greatly reduced the wavefront error so that the imaging quality reached the diffraction limit. The geosynchronous (GEO) imaging spectrometer designed by Wang H et al. [21] is based on the Offner configuration, while both the mirrors and convex grating are freeform Zernike surfaces instead of the traditional spherical surface. The introduction of the freeform surfaces can reduce the residual astigmatism induced by the long slit significantly. Yang T. et al. [22] used a point-by-point design method to create a compact freeform grating reflection imaging spectrometer. Muslimov E. R. et al. [23] made advanced spectrometers with holographic gratings on freeform surfaces. Moreau V. et al. [24] introduced ELOIS and CHIMA [25], two new instruments made of all aluminum and based on freeform diffraction gratings (FFG: freeform grating). This is a solution that makes the instruments about four times smaller than Offner–Chrisp spectrometers that do the same job.

The latest trend in push-scan imaging spectrometers [26] is for them to have a high spectral resolution, good image quality, and compact configurations. Complex blazed gratings, freeform optics, and hyper-spectral CMOS sensors are three new technologies that could be used to make useful hyper-spectral instruments in the future. Therefore, there is an urgent need to develop a new technology to solve the challenge of efficient and precise preparation of freeform gratings. This paper summarizes the effects of various grating groove shapes and structural parameters on the spectral range and diffraction efficiency of convex blazed gratings, reviews the latest progress in typical processing methods of convex blazed gratings, reviews the characterization methods and future feasible characterization methods of convex blazed gratings, and points the development trend of efficient and precise preparation of convex blazed gratings.

## 2. Functions and Applications of Convex Blazed Gratings

### 2.1. The Effect of Grating Groove Shape on the Spectral Range

**Figure 2:**
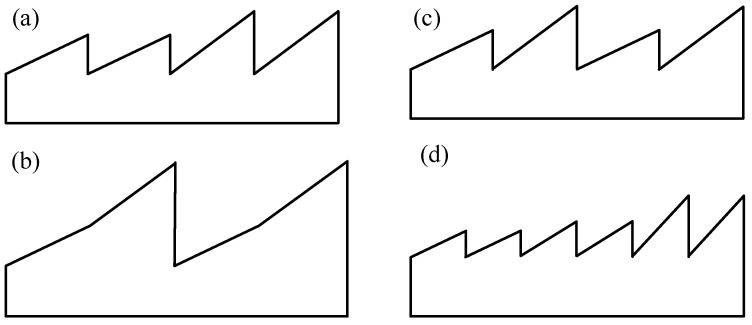
Convex dual-blaze grating. (**a**) Dual-panel blazed structure [28]. (**b**) Dual-angle blazed structure [28]. (**c**) Dual-blaze angle structure [31]. (**d**) Schematic diagram of multi-blazed grating groove structure [32].

Planar and concave gratings have well-known theories and processes for making them, while convex gratings are not as common and widely used as planar and concave gratings because they are harder to make and detect [27]. The groove shape of convex gratings can be split into two groups: Laminar gratings, also called rectangular groove gratings, are easy to make but have low diffraction efficiency, which hurts the performance of imaging spectrometers. Blazed gratings, on the other hand, are hard to make but have high diffraction efficiency, which helps imaging spectrometers perform well. P. Mouroulis [28] made two different kinds of dual-blaze convex gratings: the dual-panel blazed grating and the dual-angle blazed grating, which are shown in Figure 2a,b, respectively. A dual-panel blazed grating has two sections with distinct blaze angles, but the blaze angle is uniform in each zone, whereas a dual-angle blazed grating has a uniform groove shape throughout the whole grating surface, with each groove having two different blaze angles. Both types of gratings achieve homogeneous diffraction efficiency curves over the wavelength range of 400 nm to 2500 nm, but only the second type is immune to phase aberrations and phase abruptness caused by partitioning. ELOIS VISWIR is a compact hyper-spectral imager payload designed by Belgium’s Advanced Mechanical and Optical Systems (AMOS) and contains a 36.77 line/mm convex freeform diffraction grating. The freeform diffraction grating has the grating surface divided into two blazed regions (A and B) with blaze wavelengths of 900 nm and 1420 nm, accounting for 45% and 55%, respectively, of the effective region [29]. The broadband Offner imaging spectrometer was built by Yang F et al. [30] for asteroid detection. The spectral range of the imaging spectrometer must include the 0.4–3 μm VNIR and SWIR bands, and the convex grating surface consists of two grooved sections with distinct periods. Calcines A [31] et al. demonstrated how to design and produce gratings with more than one blaze angle (multi-blaze angle) and variable spacing, as illustrated in Figure 2c, to widen the spectral application and increase spectral resolution. Because diffraction gratings with a single blaze angle cannot cover the spectral range from 0.7 μm to 5 μm with the required diffraction efficiency, B. Sabushimike [32] proposed to design multi-blazed grating groove structures, which are shown in Figure 2d, based on the scalar diffraction theory and *f*solve optimization tool. The fabricated convex multi-blazed grating is used as the core component of the infrared hyper-spectral imager for Chandrayaan-2 lunar exploration. The grating is designed to have nine blaze wavelengths and meet the spectral range of 0.7–5 μm. B. Sabushimike [33] also proposed a method based on the MATLAB solution of the *f*solve function for the nonlinear system of equations to further reduce the spectral range covered by nine blaze wavelengths to only three blaze wavelengths, which provides significant optical and fabrication advantages. Dual-panel blazed grating can realize different spectral ranges of simultaneous imaging, while dual-angle blazed grating can further improve grating diffraction efficiency. The imaging spectral range can be effectively broadened by the introduction of multi-blaze grating.

**Figure 3:**
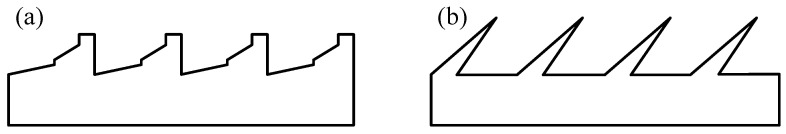
Non-triangular groove grating structure. (**a**) Convex grating with structured-groove profile [34]. (**b**) Various groove shape profiles used for echelles [35].

Scholars have also debated the spectral range effect of non-triangular groove gratings. Backlund et al. [34] of JPL used the optimal rotation angle method to create a convex grating (structured-groove grating) with a heterogeneous groove structure in 2004. The grating exhibits a pretty high and uniform diffraction efficiency distribution across the entire sunspot radiation spectral range (0.38–2.5 μm), as shown in Figure 3a. Zhang S et al. [35] examined the groove characteristics of an echelle grating with high diffraction efficiency and optimized the grating with a grating density of 79 line/mm by rigorous numerical simulations, and its structure is illustrated in Figure 3b. Although the structured-groove grating has a fairly high diffraction efficiency, it is also considerably more difficult to process.

### 2.2. Effect of Grating Structure Parameters on Diffraction Efficiency

In addition to the shape of the groove, the main structural parameters of a convex blazed grating are the grating period, grating height, grating top angle, blaze angle, base surface shape, and so on. In the process of making a convex blazed grating element, there is a certain processing error between the actual grating profile and the ideal grating profile. This is because the accuracy of the preparation method has its limits. To get the required grating diffraction efficiency, this factor should be taken into account when designing the grating structure parameters, and the designed diffraction efficiency will be higher than the required diffraction efficiency.

**Figure 4:**
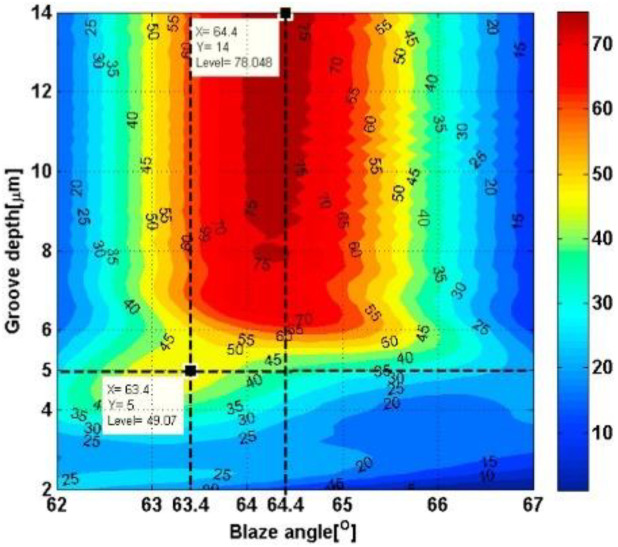
Diffraction efficiency versus grating structure size [35].

LIU Q et al. [36] used rigorous coupled-wave analysis to study how the actual groove shape of the convex blazed grating affects the diffraction efficiency. They found that when the actual grating top angle was larger than 90°, the peak diffraction efficiency dropped by a lot, but the effect on both ends of the whole band was small, and the first-order diffraction efficiency was more than 40% in the whole VNIR band. Xiong Z [37] designed the macroscopic optical structure parameters of the Offner system using traditional geometric optical theory and image quality evaluation methods. He also used rigorous coupled-wave analysis and particle swarm optimization algorithms to optimize the microstructure parameters of the convex blazed grating of the mid-wave infrared Offner imaging spectrometer. Zhang S W et al. [38] used the electromagnetic theory of grating to study how the blaze angle and apex angle affect the diffraction efficiency of a classical blazed grating in Littrow self-aligned incidence. To get the best diffraction efficiency, the effect of these two parameters on diffraction efficiency is looked at and optimized. The relationship between diffraction efficiency and grating structure size is shown in Figure 4, and the blaze angle is the key factor affecting the grating diffraction efficiency.

## 3. Fabrication Capability of Convex Blazed Gratings

This section describes the preparation technology for convex blazed gratings, covering the research outcomes of conventional processing techniques such as ultra-precision processing technology, high-energy density beam processing, and chemically assisted manufacturing processes. Electron beams, ion beams, laser beams, and other high-energy particles are examples of high-energy density beams. Current convex grating preparation techniques include electron beam lithography, holographic ion beam etching, the single-point diamond procedure, and wet etching.

### 3.1. High-Energy Density Beam Processing

#### 3.1.1. Electron Beam Lithography

**Figure 5:**
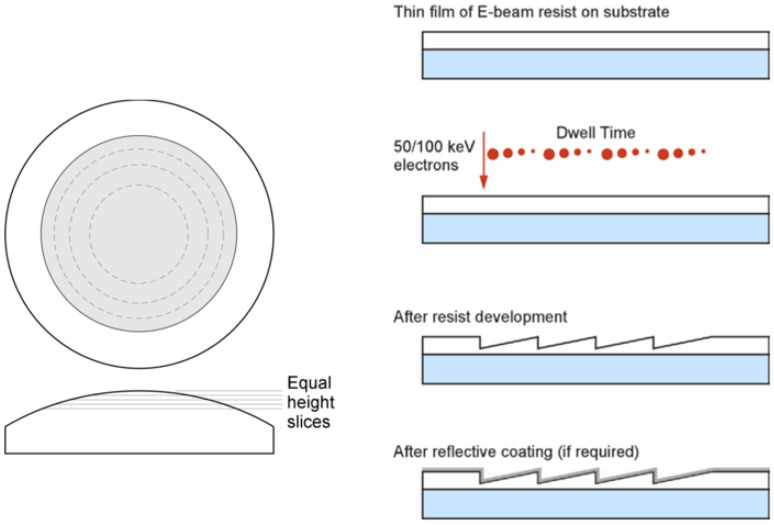
Flow of grating preparation by electron beam lithography [40].

Electron beam lithography (EBL) is a type of micromachining that uses an electron beam instead of a mask to make high-resolution patterns on a substrate. Both convex and concave diffraction gratings are needed to make concentric imaging spectrometers. Direct-write electron beam lithography has been shown to be a good way to make highly efficient blazed gratings on surfaces that are not flat. At the moment, the Jet Propulsion Laboratory (JPL) in the United States is the only place in the world that can use direct electron beam writing to make high diffraction efficiency convex blazed gratings [28]. Fujita T [39] from Osaka University in Japan wrote about a way to improve the efficiency of blazed gratings by using electron beam lithography to make transmissive blazed gratings in polymethyl methacrylate films. Measurements showed that their first-order diffraction efficiency was only 60–70% at 0.633 μm. Using the JEOL JBX-9300FS electron beam lithography system with the process schematic shown in Figure 5, the Jet Propulsion Laboratory (JPL) has produced a relative efficiency greater than 90%, with diffuse scattering and ghosting of the main diffraction order less than 5 × 10^−4^ and a zeroth-order wavefront error less than λ/4 at a wavelength of 633 nm, at a writing rate of about 1~2 cm^2^ per hour, and the technique has been used to produce a compliant grating for the Mars Imaging Spectrometer (CRISIS) with flight requirements [40]. Maker P et al. [41] prepared convex single-angle blazed and double-angle blazed gratings using the electron beam method with good wavefront quality, and a PV wavefront error of 0.2λ was observed at 632.8 nm, which is comparable to that of holographic gratings. P. Mouroulis et al. [28] used electron beam etching to fabricate convex double-angle blazed gratings and applied them to the imaging spectrometer used by the U.S. New Millennium Earth Observation Mission EO-1 in the spectral range of 400–2500 nm.

Direct electron beam technology is a complicated way to make gratings because it requires expensive and specialized hardware and software. Most of the time, it can only make convex gratings with a large radius of curvature or gratings that are close to flat, and it still cannot make non-planar features. At the same time, the direct-written point-by-point processing method is inefficient and needs very accurate equipment. It is also expensive and hard to make large areas with this method, so it cannot be mass-produced.

#### 3.1.2. Ion Beam Etching

Commercial convex gratings made by holographic ion beam etching for imaging spectrometers are mostly made in Europe, the United States, and Japan by companies such as Jobin-Yvon Inc. in France; Carl Zeiss Inc. in Germany; NASA and Richarderson Grating Laboratory Inc. in the United States; and Headwall Photonics Inc., Spectrogon Inc., and Hitachi in Japan, but the details of the process have not been made public.

Zamkotsian et al. [42,43] used sol-gel material to replicate the rectangular grating twice in UV, and then converted it into a blazed shape by low grazing angle Ar ion etching, and further made a convex grating with a period of 3300 nm and a blaze angle of 5.04° by reactive ion etching on a convex substrate with a radius of curvature of 225 mm and a reference surface diameter of 63.5 mm. This new non-planar reflection grating will be a key part of high throughput spectrometers for future space missions. Burkhardt et al. [44] from Carl Zeiss made a convex grating for the European Sentinel 5 mission’s UV-1 imaging spectrometer using the classical plane-wave symmetric holographic exposure optical path. The convex grating substrate is put in the interference field of the two parallel beams for exposure. For ion beam etching, it is important to make a holographic convex grating mask with uniform grooves. This is because holographic exposure is needed to make the grooves of the mask go all the way to the bottom of the curved substrate. Wang D. et al. [45] looked at how the light field and exposure parameters affected different spots on the surface of the photoresist substrate to make a holographic mask that can develop to the bottom of the whole convex substrate with the same recess depth to meet the needs of ion beam etching.

**Figure 6:**
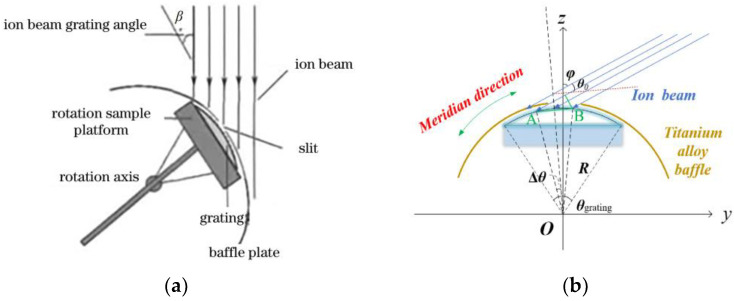
Schematic diagram of ion beam etching for making convex blazed grating. (**a**) Ion beam etching scanning stage [48]. (**b**) Oscillating ion beam etching model [52].

Due to the difficulty of making small blaze angle gratings with ion beam etching, Osterried [46] from Zeiss proposed a way to make planar blazed gratings by dip coating in 1998. Cheng Y [47] from Soochow University improved the traditional dip coating process and made a new type of dip coating device to make a convex blazed grating with a blaze angle of less than 4°. Haibin W [48] from Soochow University used Ar ion beam grazing incidence rotational scanning etching (oscillating etching method) to make a convex holographic grating with a size of 32.55 mm, a convex radius of 71.78 mm, an etched line density of 200 gr/mm, and a blaze angle of about 4.3°. By using holographic scanning ion beam etching, Liu Q et al. [49] made a convex blazed grating with a central period of 5 m, a blaze angle of about 4.3°, an etching area of 72 mm in radius, and an opening of 35 mm. Liu Q [50] also made a UV-VIS-NIR holographic double-blazed grating with a period of 833 nm using a modified holographic ion beam etching. He showed that when the two blaze angles of the dual-blaze grating were 10° to 12° and 26° to 32°, the first-order diffraction efficiency between 0.25 μm and 1 μm was more than 30%. Liu Q et al. [51] used rigorous coupled-wave analysis to study the diffraction efficiency of convex gratings (RCWA). The results showed that by controlling the blaze angle of convex gratings in the visible and near-infrared bands, the first-order diffraction efficiency can reach 40%. Above, a center period of 6.17 m, a blaze angle of about 2.9°, an antiblaze angle of about 21°, and a vertex angle of about 21° were made using holographic lithography-scanning ion beam etching technology. Experiments have shown that the first-order diffraction efficiency is more than 30% for a convex grating with a radius of about 156°, an etching area of 36.31 mm, and an aperture of 23.6 mm. At the blaze wavelength, the first-order diffraction efficiency can reach 62%. Shen C [52] from the University of Chinese Academy of Sciences made a convex blazed grating with a size of 67 mm, a curvature radius of 156.88 mm, an inscribed line density of 45.5 gr/mm, and a blaze angle of 2.2° using the holographic-swing ion beam etching method. Its peak diffraction efficiency is in the wavelength range of 900 nm–2500 nm. The ion beam etching method is shown in Figure 6.

Holographic ion beam etching is the most maturely studied and widely used method for preparing convex gratings, which has the advantages of low cost, controllable groove shape, and no ghost lines. However, the holographic method has no advantage in the fabrication of gratings with low inscribed line density [48], while the axial limitation in the ion beam etching device and the need for beam modulation limit its ability to produce blazed gratings on freeform substrates.

### 3.2. Ultra-Precision Machining Technology

Most of the techniques for making gratings that are currently available, such as direct ruling, holographic ion beam etching, or electron beam lithography, work best for grating surfaces with simple substrates, such as flat surfaces or spherical surfaces with a large radius of curvature. The high machining accuracy of the diamond turning process has shown that it can be used to make advanced functional optical surfaces like discontinuous microstructures and freeform surfaces [53,54]. Using the inexpensive single-point diamond turning (SPDT) method, Advanced Machine and Optical Systems (AMOS) in Belgium showed that freeform gratings (FFG), which are blazed gratings on surfaces with no rotational symmetry, are possible [19]. High-quality freeform blazed grating elements made with a diamond can even be used instead of electron beam lithography to make holograms [55,56].

The fast tool servo-assisted turning process dynamically adjusts the depth of cutting by additional tool motion to machine more uniform multilayer and freeform diffractive optics. A nano-fast tool servo system (nFTS) was proposed by Brinksmeier et al. [57] to adjust the depth of cutting in the nanometer range for holographic pattern machining. XinQuan Zhang et al. [58] proposed a five-axis swinging-rotating diamond shaping (SDS) process that allows precise tool swinging for Fresnel lens machining on rollers with an optically effective surface roughness of less than 10 nm. Both techniques demonstrate that ultra-precision machines possess a high degree of flexibility to machine parts with non-rotationally symmetric features.

**Figure 7:**
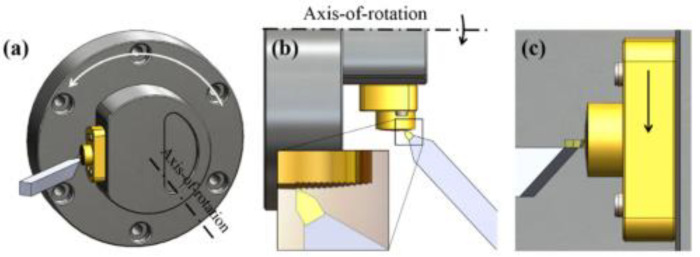
Schematic diagram of the multi-degree-of-freedom diamond tool holder [62]. (**a**) three-dimensional view, (**b**) top view, (**c**) front view.

Meier A [59] investigated the performance of three nickel-silver alloys N37, N22, and N31 in machining diffraction microstructures with a nano-fast tool servo system (nFTS) and found that alloy N31 produced more surface defects but had better structural accuracy and the lowest surface roughness for machining diffraction optics. Ann-Katrin Holthusen [60] et al. employed a nano-fast tool servo system (nFTS) to produce a blazed grating structure with a height of 0.96 μm, an optically effective surface length of 8.36 μm, and a blaze angle of 6.6° on a nickel-silver alloy N31 workpiece. By coordinating the multi-axis motion of ultra-precision machine tools and precisely managing the position of tool-workpiece contact according to the geometric needs of convex gratings, a grating structure with a changeable grating pitch may be manufactured. ChaBum Lee et al. [61] created a blazed grating nickel mold with the following blazed grating dimensions: a grating period of 2.0 μm, grating height of 0.2 μm, and blaze angle of 5.86° using the diamond tool interferometric ruling method. Di Xu et al. [62] machined a convex grating with a changeable grating pitch on a brass C46400 substrate using a Moore Nanotechnology 350 FG ultra-precision five-axis machining tool. The grating density was 300 lines/mm, and the surface roughness on the grating blazed surface was around 10–15 nm RMS and 30–45 nm·Rz, as shown in Figure 7, the design is acceptable for the 500–1100 nm spectrum range. The above studies demonstrate the possibility of ultra-precise turning of blazed gratings on metallic convex spherical substrates.

Chun-Wei Liu et al. [63] employed the diamond turning method to investigate the effect of shape design, grating period, and cutting speed on mold machining performance on brass rollers. Diamond turning for mold materials is a feasible way to ensure continuous mass production of subwavelength gratings. Tan N Y J et al. [64] processed the high-curvature radial grating and freeform surface grating on the brass workpiece using the Continuous Rotating Freeform Shaping (CRFS) algorithm in conjunction with the slow slide servo system. The average grating period is 12.5075 μm, the average blaze angle is 8.25 ± 0.01°, the surface roughness Sa may reach 0.015 μm and 0.018 μm, respectively, and the profile deviation is less than 0.3%. The substrate shape of convex blazed gratings is further expanded from simple spherical surfaces to ellipsoidal surfaces, enhancing the possibility of complex curved blazed gratings with single-point diamond turning.

De Clercq C [19] utilized a five-axis ultra-precision lathe and a single-point diamond tool to machine freeform gratings on a chemically plated NiP surface of an aluminum 6061T6 substrate with a diameter of 35 mm and a curvature radius of 80 mm. The roughness measured in a single groove was close to 4 nm RMS with a blaze angle of 1.82°. Zhizhong Z et al. [65], based on a five-axis ultra-precision single-point diamond lathe cutting process, calculated the machining deviations of the diamond head movement error, the standard deviation range of the movement interval, and the grating inscription position, and a convex blazed grating with a substrate of 6061 aluminum, a curvature radius of 70 mm, a ruling density of 60 lines/mm, and a diameter of 52 mm was successfully developed, with a maximum relative diffraction efficiency greater than 80% in the spectral range of 1000–2500 nm. Graham C et al. [66] introduced an all-aluminum, rugged, lightweight, freeform-based near-infrared hyper-spectral moisture sensing imager (FYMOS). The design uses a custom freeform blazed grating made on a five-axis ultra-precision diamond machine to get the best optical performance while keeping the size and weight as small as possible. Bourgenot C et al. [67] from Durham University in the UK wrote about the technical challenges and progress made in making convex blazed gratings on an ultra-precision five-axis Moore machine. They also talked about how the gratings are used as optical pupil reflectors in an integrated grating imaging spectrometer (IGIS) integral field unit prototype. In a comparison of freeform gratings machined on RSA 6061 and RSA 443 substrates chemically coated with NiP surfaces with an average roughness of 2.5 nm RMS per blazed surface, Bourgenot C et al. [68] discussed new opportunities for freeform gratings machined with diamond and identified NiP as the substrate material with the best roughness and profile quality. Li H et al. [69] used a four-axis ultra-precision machining system for turning a convex spherical blazed grating with a curvature radius of R = 41.104 mm, a substrate diameter of 14 mm, a grating density of 53.97 lines/mm, and a blaze angle of about 3.8°. The blazed surface roughness is better than Ra2 nm. The above research shows that ultra-precision machining technology can achieve efficient and high-precision machining of freeform blazed gratings.

All freeform gratings based on three-mirror configuration (Offner type) spectrometers are machined using single-point diamond turning (SPDT) as part of the ESA Technical Research Program (TRP) [70]. Diamond-machined gratings are the best option in some applications, despite the fact that they cannot yet match holographic gratings in terms of stray light. It is obvious that photoresists on the surface of holographic gratings are not appropriate in imaging systems where the mechanical characteristics of the substrate need to be enhanced for thermal insulation purposes or for use in harsher environmental conditions. The manufacturing of convex gratings by single-point diamond ultra-precision cutting is a new processing approach developed in recent years that allows the combining of slow slide servo (SSS) systems to produce gratings on a range of substrate surface shapes in a controlled and synchronized manner [71,72]. This not only enables the execution of repeated passes for improved workpiece finishing but also the production of gratings on more difficult and irregular surfaces. Because ultra-precision five-axis machines have almost no limitations in the machining of substrate surface shapes, can machine blazed gratings on high-curvature surfaces, and the ease of precise control of blaze angles with this technique provides a greater advantage in producing high diffraction efficiency gratings, diamond-machined gratings become a cost-effective and viable alternative [73].

### 3.3. Chemically Assisted Manufacturing Process

X-ray diffraction gratings operate at very shallow incidence angles due to the critical angle limitation, so the blaze angle of faceted or blazed gratings should be very small, ranging from a few degrees for extreme ultraviolet (EUV) and soft X-ray spectrometers to a fraction of a degree for hard X-ray applications (e.g., X-ray free electron lasers) [74]. For ultra-precise processing techniques and holographic ion beam etching, conventional approaches can produce gratings with reasonably deep grooves and moderate blaze angles that are of good quality. However, making triangular grooves with small blaze angles (less than 5°) is challenging for both techniques. Voronov D L et al. [75,76] from Lawrence Berkeley National Laboratory, USA, demonstrated a silicon-based technique for anisotropic etching, which aimed to fabricate X-ray diffraction gratings with ultra-low blaze angles with high efficiency and precision, thereby extending grating technology to the X-ray wavelength range from 1 nm to 0.1 nm. The gratings were fabricated using a set of nanofabrication techniques including e-beam lithography, nanoimprint, plasma etch, and anisotropic wet etching [77]. For X-ray diffraction gratings, only this technique can be used for processing.

### 3.4. Other Feasible Processes

X-ray lithography to fabricate convex gratings first requires a grayscale mask with a triangular pattern on a gold-plated silicon nitride film, and then uniformly and linearly scanning the substrate masked by the mask with X-rays. If the exposure characteristic curve of the photoresist is linear, the blazed groove shape can be obtained. P. Mouroulis et al. [78] from JPL used X-ray grayscale exposure lithography to produce convex blazed gratings with an aperture of 20 mm, a curvature radius of 100 mm, a grating period of 20 μm, and a spectral range of 0.4 μm to 1.1 μm. Their relative diffraction efficiency at the blaze wavelength can reach 88% with a wavefront quality less than λ/8. However, this method can only make sinusoidal gratings, which diffraction efficiency is much lower than that of blazed gratings [79].

**Figure 8:**
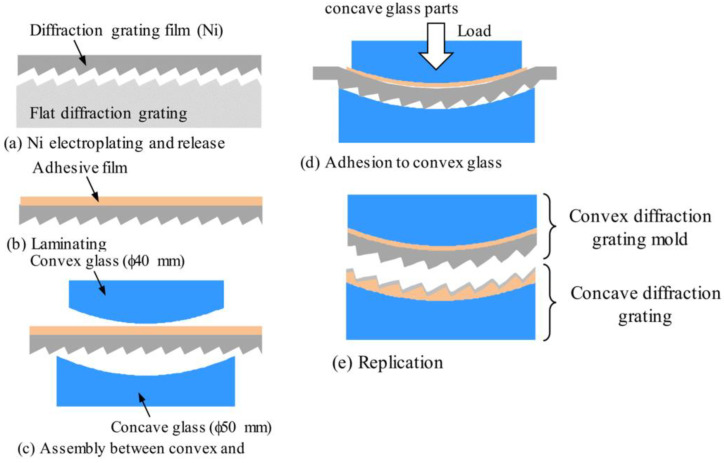
Process flow diagram for making convex and concave gratings by 2D bending method [81].

Hitachi [80] came up with a way to turn a flat grating substrate into a convex grating by bending it. The method starts by using a semiconductor process to make a planar grating with a precise groove shape on a silicon substrate. The groove shape is then copied onto a flexible amorphous material (like resin) or a metal film substrate, which is then bent and mounted on a pre-designed substrate with a fixed curve. This method can produce both convex and concave gratings and has the advantage that the curvature of the substrate is not limited and is determined only by the design. Aono T et al. from Hitachi [81] also proposed a two-dimensional bending technique to obtain both convex and concave gratings of 20 mm in length and 20 mm in width with a curvature of 100 mm, as shown in Figure 8. However, this technique is limited not only by the preparation quality of the concave grating but also by the quality of the release. So, the processing accuracy is relatively low and cannot meet the requirements.

**Figure 9:**
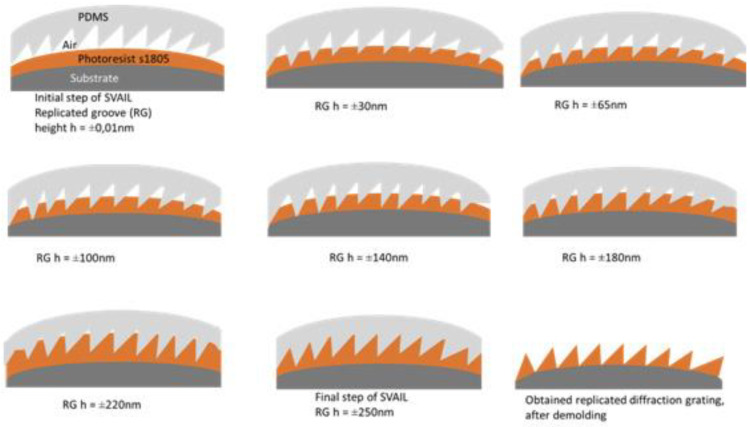
Evolutionary representation of replication groove (RG) height during SVAIL replication [82].

Horugavye G et al. [82] from the University of Liege, Belgium, reported a method to achieve diffraction grating replication on a convex substrate using a solvent vapor-assisted imprinting lithography (SVAIL) process with a convex grating size of 12.7 mm × 12.7 mm × 6 mm, grating density of 600 line/mm, blaze angle of 8°37′, and blaze wavelength of 500 nm, and the main steps of the SVAIL replication process are shown in Figure 9.

However, these methods are not cost-effective, have low preparation efficiency, are only suitable for small-aperture optical components of about 20 mm, and are not commonly used processes.

## 4. Adaptability of Manufacturing Processes at Different Scales (Summary of Processing Limits)

According to Table 1, each of the aforementioned convex grating preparation techniques offers benefits and drawbacks that are unique. We analyze the best fabrication techniques for different convex blazed gratings by comparing the advantages and disadvantages of each method according to the usage requirements of imaging spectrometers (high diffraction efficiency, large relative aperture, and low stray light) and the actual preparation conditions.

## 5. Quality Characterization of Convex Blazed Gratings for Hyper-Spectral Imaging Spectrometer

The processing quality of a convex blazed grating, which must be defined in terms of shape, diffraction effectiveness, polarization sensitivity, scattering, and operating spectrum range, directly affects the imaging performance and quality of a hyper-spectral imager. In the following sections, two methods of contact measurement and non-contact measurement will be used to analyze the current quality characterization methods for convex blazed gratings as well as the potential future methods.

### 5.1. Contact Measurement

**Figure 10:**
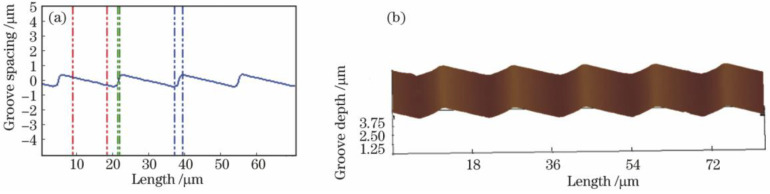
Convex grating cross-sectional profile. (**a**) Surface profile of convex grating [65]. (**b**) AFM morphology of convex grating [65].

Atomic force microscopy’s contact mode is usually used to look at the profile integrity and blaze angle of convex blazed gratings [45]. Because the AFM has a small field of view, this measurement can only be done on convex gratings with a grating period of less than 100 μm. In studies [65,83], AFM was used to get a local profile of the grating, which is shown in Figure 10. This lets the grating period, blaze angle, and other parameters be measured.

### 5.2. Non-Contact Measurement

#### 5.2.1. Direct Wavefront Measurement Method

**Figure 11:**
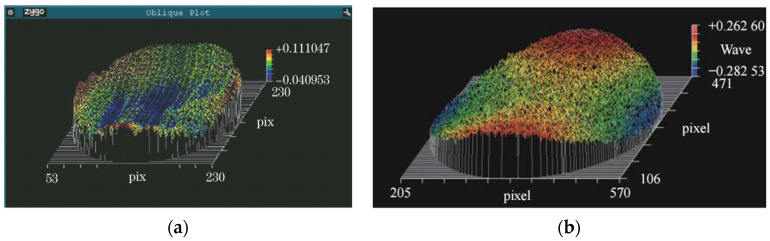
Convex grating zeroth-order optical wavefront morphology. (**a**) Wavefront morphology [65]; (**b**) Wavefront morphology [84].

The phase difference of the grating diffraction wavefront determines the quality of the spectral lines and the resolution of the grating. The grating diffraction wavefront is often measured by comparing the actual wavefront to a standard plane or sphere. In the study [65], an interferometer (ZyGo, model VerifireTM) was chosen to test the quality of the grating surface using convex grating zeroth-order light. The test results are shown in Figure 11a, where the peak-to-valley (PV) value of the surface profile is 0.152λ and the root mean square (RMS) is 0.027λ where wavelength is 632.8 nm. The researchers in [84] used the Zygo interferometer to conduct an interference test on the zeroth-order diffracted light of the convex blazed grating and the surface error peak-valley value (PV) of the grating surface was 0.545λ_test_ and the root mean square value (RMS) was 0.085λ_test_, among which the test wavelength λ_test_ was 632.8 nm, as shown in Figure 11b.

**Figure 12:**
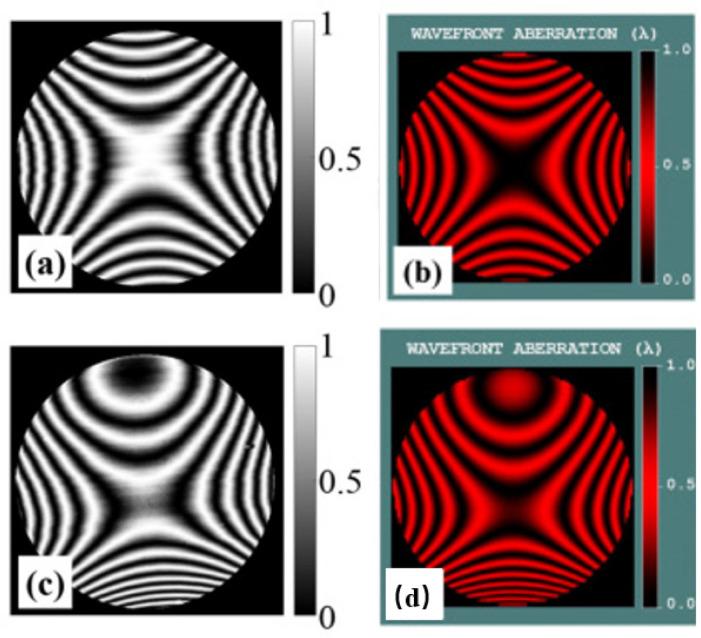
On the left, (**a**,**c**) are the experimentally obtained interferograms of the equal-along-projection grating and the equal-along-arc grating. On the right, (**b**,**d**) are the expected interferograms of the equal-along-projection grating and the equal-along-arc grating [62].

A phase-shifted Fizeau interferometer (Zygo Verifire) can be used to take direct measurements of diffraction wavefronts at different levels of diffraction [62]. To describe the quality of processing of convex gratings, the study [62] compared the expected interferograms from optical design software that simulates the test conditions with those obtained by direct wavefront measurement methods, as shown in Figure 12.

#### 5.2.2. Nulling Wavefront Metrology Method

**Figure 13:**
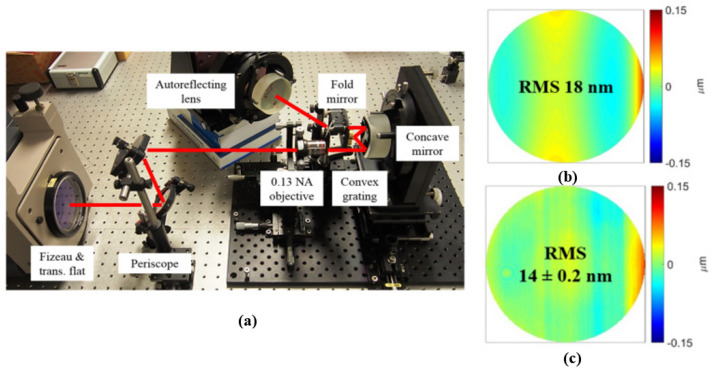
(**a**) Experimental setup for nulling interference test coupled to a phase-shifted Fizeau interferometer. (**b**) Designed expected nulling wavefront. (**c**) An example of the nulling wavefront obtained in the experiment [62].

The nulling wavefront metrology method is detected using the experimental setup shown in Figure 13a, utilizing a concave spherical mirror placed concentrically with the convex grating substrate, which acts as an aperture diaphragm. The numerical aperture of the object of F/3.8 was chosen in the study [62] to match the numerical aperture of the designed imaging spectrometer. At the final zero position, 100 consecutive phase measurements were performed using a phase-shifted Fizeau interferometer [85]. To reduce the effects of vibration noise and air turbulence noise on the detection results, these measurements were then roughly split into five groups and averaged within each group. The calculated RMS of the nulling wavefront over these five data sets was 14 ± 0.2 nm. Figure 13c shows an example of one of these measurements, and the machined convex gratings were compared to the expected RMS of the nulling wavefront in Figure 13b to see if the design requirements were met.

#### 5.2.3. Nonnull Interferometric Testing

Eliminating the influence of the groove pattern from the measured data is a major advancement for spherical gratings with an uncertain groove distribution. A spherical grating’s diffraction wavefront includes intrinsic wavefront contributions from the self-reflecting test equipment in addition to manufacturing-related wavefront imperfections. The magnitude of the latter is influenced by the spherical substrate and the groove pattern. A nonnull interference method for spherical grating interferometric detection was put forth in the study [86] by analyzing the geometric aberration of spherical gratings. This method involves a secondary measurement under Littrow conditions with various diffraction orders in the case of unknown spherical stripes in order to obtain the wavefront error caused by fabrication defects only. The experimental sample was a concave spherical grating with a NA value of 0.13 and a grating density of 200 lp/mm, and a diffraction wavefront error of 0.018λRMS was obtained.

#### 5.2.4. Grating Profile Accuracy Detection and Surface Shape Error Characterization

Grating surfaces that are smooth and can detect sharp edges are a requirement for high-performance optical applications. When the period of the convex grating is large and inspection by AFM is not appropriate, the geometry of the blazing structure can be measured and analyzed using white light interferometry. On each grating cell of the grating cross-sectional profile A and B, the structure height (h), the length of the optically effective surface (s), and the blaze angle (k) can be measured. The mean and standard deviation of the observed values of each parameter can then be calculated [60,87]. The roughness measured in a single groove was close to 4 nm RMS, which is close to the limit of non-straight optical surface roughness for single-point diamond turning machining. The study [19] also described the grating blazed surface roughness using white light interference and measured the roughness at three locations (center and both sides) on a freeform grating. A laser confocal microscope can also be used to determine the cross-sectional profile and blazed surface roughness of convex gratings. Based on this inspection method, the surface roughness (Sa) of machined convex ellipsoidal gratings measured in the study [64] can reach approximately 0.015 μm and 0.018 μm.

#### 5.2.5. Stitching Measurements of Freeform Diffraction Microstructures

The study [88] uses the coherent correlation interference (CCI) principle of the Taylor Hobson optical profilometer to perform multiple measurements in grating mode to precisely align the surface by using the same reference as in the manufacturing process. A 20× objective is selected to scan the surface and consider the total number of measurements in the area required to measure the diffracted structure. The surface shape as well as the diffraction structure is obtained by overlapping the measured values with 20% of the stitching. The stitched surface contours obtained by the optical profilometer are unprocessed and unfiltered to isolate usable contour information, and direct comparison of the original to the design surface contours is very challenging due to alignment issues. To solve this problem, the raw measurements are processed to extract information about the underlying shape and diffraction structure separately. Stitching measurements [89] are an effective way to obtain freeform diffraction microstructure full-scale shape and microstructure unit size information, which is important for the full-scale shape characterization of convex blazed grating elements.

### 5.3. Diffraction Efficiency Test

The diffraction efficiency of a grating is an important measure of how well it works. It is usually split into two types: absolute diffraction efficiency and relative diffraction efficiency. In terms of spectroscopy, the absolute diffraction efficiency *η*(*λ*,*m*) of a grating is defined as the ratio of the diffracted luminous flux *E*_g_(*λ*,*m*) of the grating received by the detector to the luminous flux *E*_0_(*λ*,*m*) incident on the surface of the grating at a particular wavelength *λ* and spectral diffraction order *m*.

(1) $$\eta_{}=\frac{E_{g}(\lambda ,m)}{E_{0}(\lambda ,m)}$$

The relative diffraction efficiency *η*′(*λ*,*m*) of the grating is defined as the ratio of the diffracted luminous flux *E*_g_(*λ*,*m*) of the grating received by the detector to the reflected luminous flux *E_r_*(*λ*,*m*) of a standard reflector of the same aperture for a given wavelength *λ* and diffraction order *m*.

(2) $$\eta^{\prime }=\frac{E_{g}(\lambda ,m)}{E_{r}(\lambda ,m)}$$

The purpose of distinguishing between absolute and relative diffraction efficiency is that the former can help spectroscopy instrument manufacturers to calculate the light energy utilization of the instrument, while the latter can help grating manufacturers to evaluate and improve the manufacturing process. From the definition of grating diffraction efficiency, it can be seen that the way to measure grating diffraction efficiency is to measure the incident luminous flux at a certain wavelength on the working order of the grating to be measured and the reflected luminous flux of the accompanying coating. The relative diffraction efficiency of the grating can be found using Equation (2). The absolute diffraction efficiency of the grating can be found by multiplying this value by the reflectivity of the coating that goes with it. Therefore, the measurement of grating diffraction efficiency is an indirect measurement.

**Figure 14:**
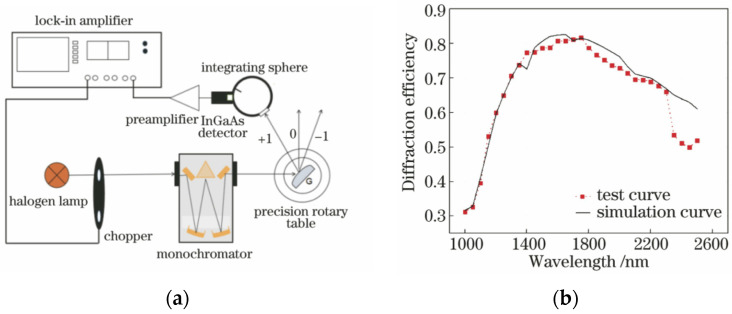
Test setup and curve for diffraction efficiency of convex grating. (**a**) Schematic diagram of the grating diffraction efficiency test setup [65]. (**b**) Convex blazed grating diffraction efficiency curve [65].

So far, the relative diffraction efficiency of gratings has been measured in three different ways: the line spectrum method, the continuous scan method, and the Fourier transform method [90]. Because the theoretical diffraction efficiency curve of a convex grating is smoother in its operating band, the researchers of study [91] chose the line spectrum method to measure the diffraction efficiency. The line spectral method has some benefits, such as a high level of wavelength accuracy and a simple structure. However, the measurement data are not continuous, and the diffraction efficiency characteristic curve cannot be directly obtained. The grating has a strong polarization effect, and its efficiency depends on the direction of polarization. This means that the efficiency is different for polarized light that is perpendicular to the groove and for polarized light that is parallel to the groove. This is why the efficiency is measured in natural light. In the study [65], the diffraction efficiency of a grating was measured at a given wavelength and diffraction order by using a reflector with the same aperture as the grating being tested and coated with the same gold film as a reference, recording the reflected energy at the same wavelength, and figuring out the ratio of the two measurements. The grating diffraction efficiency is measured using a device such as that shown in Figure 14a, which consists of a halogen light source, a monochromator, a grating holder, an integrating sphere photometric device, and a lock-in amplifier. The focal length of the grating monochromator is 300 mm, the grating inscription density is 300 lines/mm, and the spectral range is set from 1000 to 2500 nm. The slit of the monochromator is 0.05 mm, and the spectral bandwidth is about 0.6 nm, which is smaller than the theoretical bandwidth of the convex grating. The plane mirror and the convex grating were placed on a precision rotary table. The spectral range was scanned in steps of 50 nm, and the energy values *E_λ_* were recorded separately. The diffraction efficiency curve of the convex blazed grating is shown in Figure 14b.

## 6. Conclusions and Potential for Future Development

Efficient and precise fabrication of convex blazed gratings is of great importance for scientific and industrial applications. In this paper, we reviewed several typical methods for processing convex blazed gratings and present the basic principles and methods for their further development, as well as their advantages and disadvantages. In particular, the structure and properties of various convex blazed gratings fabricated using these methods were presented to specify their respective limitations and feasibility. However, considering that different methods exhibit different performances in different applications, it would be inaccurate to use the same criteria to evaluate a particular index. In order to select the appropriate technology for the production of convex blazed gratings, the capacity and limitations of each method should be compared with the key performance aspects of the intended application.

One of the challenges for future Earth observation spectroscopy instruments is to accommodate larger spectral ranges with better spatial and spectral resolution. A second challenge is the improvement in temporal sampling, which is driving the technology toward smaller satellite platforms and ultra-compact payloads. The design of imaging spectrometers requires finding the best compromise between form factor, spatial resolution, and polarization sensitivity to further miniaturize the spectrometer while maintaining good sensitivity and resolution. The application of freeform gratings offers the possibility of achieving the best compromise. Freeform gratings were developed to provide an additional parameter in the optimization of optical designs to reduce keystone and smile in spectrometers. Although this solution may increase the complexity of sophisticated optical components such as gratings, it has proven to be a key component in minimizing the number of optical components. Therefore, the research progress in the preparation and characterization of convex blazed gratings for hyper-spectral imaging spectrometers can be summarized as follows.

(1) Freeform blazed gratings have broader application prospects than planar gratings.
(2) The technology for efficient ultra-precise preparation of freeform blazed gratings needs to be developed.
(3) Ultra-precision machining technology is applicable to the processing of high-curvature freeform surface blazed gratings.
(4) Stitching measurements are essential for the complete characterization of machining accuracy of freeform blazed gratings.

## Figures and Tables

**Table 1:** Comparison of several common fabrication methods for convex gratings.

Preparation Method	Ultra-Precision Diamond Turning	Ion Beam Etching	E-Beam Direct Writing	X-ray Lithography
Spectral range	Visible-LWIR	UV-NIR	Visible-NIR	Visible-NIR
Convex vector height	Almost unlimited	Practically unlimited	Less than 4 mm	Meet the production of thick line grating
Base dimension	Small	Large	Small	Large
Grating period	Low reticle density	High reticle density	High reticle density (greater than 1 μm)	High reticle density (greater than 3 μm)
Grating groove shape	Arbitrary groove shape	Sinusoidal, rectangular, triangular groove shape	Arbitrary groove shape	Arbitrary groove shape
Diffraction efficiency	High (high requirements for machining accuracy)	High (technological difficulty)	High	High
Stray light	Higher	Low	Less than 5 × 10^−4^	Less than 10^−4^
Roughness	Less than 10 nm	Low	Low	Low
Wavefront quality	Low	High	High	High
Manufacturing cost	Lower	Higher	High	High
General material	Al, Cu, NiP, and other metal materials	Fused quartz, RB-SiC, GaAs, PMMA, K9	Silicon, glass	Silicon nitride
